# Gene Expression Profiling Identifies Downregulation of the Neurotrophin-MAPK Signaling Pathway in Female Diabetic Peripheral Neuropathy Patients

**DOI:** 10.1155/2017/8103904

**Published:** 2017-08-16

**Authors:** Lin Luo, Wen-Hua Zhou, Jiang-Jia Cai, Mei Feng, Mi Zhou, Su-Pei Hu, Jin Xu, Lin-Dan Ji

**Affiliations:** ^1^Zhejiang Key Laboratory of Pathophysiology, Medical School of Ningbo University, Ningbo 315211, China; ^2^Laboratory of Behavioral Neuroscience, Ningbo Addiction Research and Treatment Center, Medical School of Ningbo University, Ningbo 315211, China; ^3^Department of Preventive Medicine, Medical School of Ningbo University, Ningbo 315211, China; ^4^Department of Research and Teaching, Ningbo No. 2 Hospital, Ningbo 315010, China; ^5^Department of Biochemistry, Medical School of Ningbo University, Ningbo 315211, China

## Abstract

Diabetic peripheral neuropathy (DPN) is a common complication of diabetes mellitus (DM). It is not diagnosed or managed properly in the majority of patients because its pathogenesis remains controversial. In this study, human whole genome microarrays identified 2898 and 4493 differentially expressed genes (DEGs) in DM and DPN patients, respectively. A further KEGG pathway analysis indicated that DPN and DM share four pathways, including apoptosis, B cell receptor signaling pathway, endocytosis, and Toll-like receptor signaling pathway. The DEGs identified through comparison of DPN and DM were significantly enriched in MAPK signaling pathway, NOD-like receptor signaling pathway, and neurotrophin signaling pathway, while the “neurotrophin-MAPK signaling pathway” was notably downregulated. Seven DEGs from the neurotrophin-MAPK signaling pathway were validated in additional 78 samples, and the results confirmed the initial microarray findings. These findings demonstrated that downregulation of the neurotrophin-MAPK signaling pathway may be the major mechanism of DPN pathogenesis, thus providing a potential approach for DPN treatment.

## 1. Introduction

Diabetes mellitus (DM) has become one of the largest global public health problems of this century. According to the World Health Organization global report on diabetes, over 8.5% of the global population, approximately 422 million adults, have diabetes [1]. Diabetes of all types can lead to complications in many parts of the body and can increase the overall risk of premature death. Potential complications include cardiomyopathy, vasculopathy, neuropathy, retinopathy, nephropathy, dermatopathy, and encephalopathy [2, 3]. Diabetic peripheral neuropathy (DPN) is characterized as a chronic symmetrical, progressive disorder with early symptoms of pain, allodynia, and paresthesia, and it affects approximately 50% of people with considerable morbidity, mortality, and diminished quality of life [4]. Despite the development of diagnostic methods and therapeutic modalities, DPN is still not diagnosed or managed properly in most patients [5].

Over the past decade, there have been many achievements in understanding the pathogenesis of DPN. However, controversy remains because of a multifactorial etiology involving both metabolic and vascular factors [6]. Chronic hyperglycemia used to be regarded as the major factor in the initiation of various metabolic events underlying DPN. Results from the Diabetes Control and Complication Trial (DCCT) support the hypothesis that DPN develops as a result of increased blood glucose and have suggested that treating hyperglycemia in type 1 DM can markedly decrease the incidence of DPN, by up to 60 to 70% [7]. However, more recent studies have reported that intensive glucose control in patients with type 2 DM has no significant effect on the development of DPN [8]. Over 40% of type 2 DM patients develop DPN despite good glucose control, thus suggesting that hyperglycemia is only one of the many key events that cause nerve and microvascular injury [9]. Nevertheless, hyperglycemia has effects on several major, well-characterized biochemical pathways that include activation of the polyol pathway [10], formation of advanced glycation end products (AGEs) and their receptors [11], activation of protein kinase C (PKC) [12] and inducible nitric oxide synthase [13], increased poly(ADP-ribose) polymerase (PARP) activity [14], and elevated inflammation [15]. Furthermore, oxidative stress and mitochondrial dysfunction [16], hypoxia and ischemia [17], elevated cytokines [18], and deficiencies in neurotrophic factors [19] also play significant etiologic roles in DPN. Despite advances in delineating the etiology of DPN, few effective therapies exist to manage, delay, or prevent the development of painful DPN. Therefore, identifying precise mechanisms and related therapeutic drugs remains paramount.

Systems biology approaches, such as whole genome expression profiling, may provide new insights into the molecular mechanisms of DPN without preset bias. However, previous studies have identified global transcriptomic changes only in animal models [20]. In the present study, we used high-throughput genome-wide expression microarrays to identify alterations in the transcriptome, both common and distinct, between type 2 DM and DPN. The results may provide relevant information for the future development of new mechanism-based diagnostics and therapies.

## 2. Material and Methods

### 2.1. Study Design

**Figure 1:**
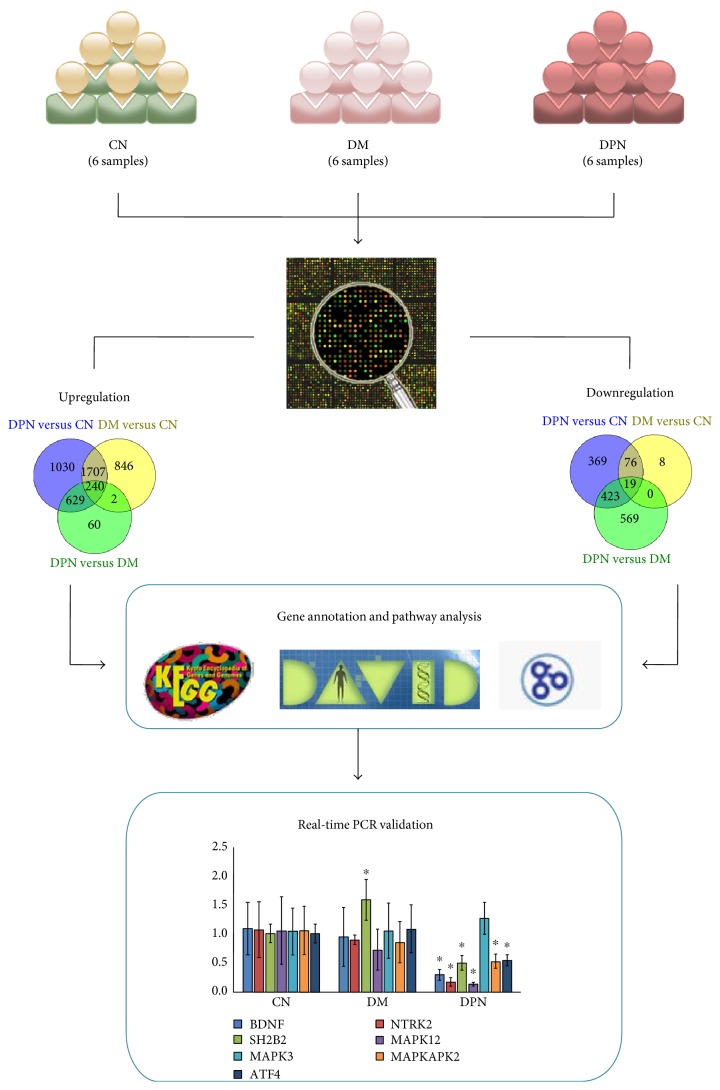
The workflow of the study. The asterisks represent statistical significance (*P* < 0.05) compared with the control group.

A workflow was designed to identify candidate genes whose expression levels may differentiate among healthy controls (CN), DM patients, and DPN patients (Figure 1()). After obtaining three individual differentially expressed gene (DEG) datasets (DM versus CN, DPN versus CN, and DPN versus DM), we used a Venn diagram to identify the shared and distinct gene expression changes between DPN and DM. Subsequently, several gene annotation databases were used to identify the potential biological functions and signaling pathways involved in DPN. Finally, DEGs of interest were further validated in an independent sample set.

### 2.2. Participants

The participants were recruited from hospitals affiliated with Ningbo University from April 2015 to June 2016. The healthy controls had no family history of diabetes or neurologic disorders, exhibited normal glucose tolerance, and were free of any major chronic diseases. Type 2 DM cases were defined as meeting at least one of the following criteria: fasting plasma glucose ≥7 mmol/L, 2 h plasma glucose after oral glucose tolerance test (OGTT) ≥ 11.1 mmol/L, and use of glucose-lowering drugs or physician-diagnosed diabetes. Diabetic peripheral neuropathy was diagnosed by a positive assessment through neurologic examinations and nerve conduction studies, as previously described [20]. In brief, the criteria for DPN were as follows: (1) confirmed type 2 DM patients; (2) decreased sensation and positive neuropathic sensory symptoms (including pricking, burning, stabbing, or aching pain) in the toes, feet, or legs; (3) decreased distal sensation and unequivocally decreased or absent ankle reflexes; (4) and abnormal motor and sensory nerve conduction. Patients with type 1 DM, cardiovascular diseases, previous history of neurologic disorders, peripheral vascular occlusive disease, autoimmune disease, or any other possible causes of peripheral neuropathy were excluded from all groups. Patients with any clinically observable diabetic complications were excluded from the DM group. The DPN patients involved in the present study were all new cases without any antineuropathy medication. Patients with any other major acute or chronic complication associated with diabetes or vitamin B12/folic acid deficiencies were excluded from the DPN group. The protocol of this study was approved by the medical ethics committee of Ningbo University. The health records and blood samples (2 mL) of the participants were collected after informed written consents were provided by the subjects. A total of 6 healthy controls, 6 DM patients, and 6 DPN patients were recruited for the microarray analysis. In the validation stage, another 26 healthy controls, 26 DM patients, and 26 DPN patients were included. An overview of the clinical and demographic characteristics of the participants can be found in Table 1.

### 2.3. RNA Preparation

Total RNA was isolated from peripheral blood mononuclear cells (PBMCs) by using a TaKaRa MiniBEST Universal RNA Extraction Kit according to the manufacturer's instructions. The RNA quality was determined on the basis of an optical density (OD) 260/280 ratio ≥ 1.8 and OD260/230 ratio ≥ 2.0 using a NanoDrop ND-1000 spectrophotometer. RNA integrity was determined using an Agilent 2100 Bioanalyzer. The intensity of the 18S and 28S rRNA bands was examined on a 1% formaldehyde-agarose gel. RNA samples with an RNA integrity number (RIN) of ≥6.0 and 28S/18S > 1.5 were subjected to microarray analysis.

### 2.4. Microarray Analysis

The microarray analysis was performed by BGI Inc., including RNA amplification, probe labeling, hybridization, and data extraction. Briefly, aliquots (100 ng) of total RNA were amplified and transcribed into fluorescent Cy5-labeled antisense RNAs (aRNAs) by using a OneArray Amino Allyl aRNA Amplification Kit (Phalanx Biotech, San Diego, CA) according to the manufacturer's instructions. The Cy5-labeled aRNAs were fragmented and then hybridized to the Human Whole Genome OneArray Version 6.0 (Phalanx Biotech, San Diego, CA). Nonspecific binding targets were washed out three times. The arrays were scanned by an Agilent G2505C Microarray Scanner (Agilent Technologies, Wilmington, DE). The fluorescence intensities of each spot were analyzed with Feature Extraction software (Agilent Technologies, Wilmington, DE). Overall, 18 microarray chips were analyzed in this study.

### 2.5. Statistical Analysis of the Microarray Data

The microarray data were normalized using the R/Bioconductor Limma package [21]. An empirical Bayes model was used to compare DEGs between groups, and the criteria for DEGs were FDR < 0.05 and |log_2_ (ratio)| ≥ 1. Hierarchical clustering was performed to visualize distinguishable gene expression profiles among the samples. The gene annotation and biological interpretation of the identified DEGs were performed using the Database for Annotation, Visualization, and Integrated Discovery (DAVID) v6.8 [22]. Biological functions, represented by Gene Ontology terms (http://geneontology.org/) and Kyoto Encyclopedia of Genes and Genomes pathways (http://genome.jp/kegg/), were deemed significant at a Benjamini-Hochberg-corrected *P* < 0.05. The R/Bioconductor Pathview package was used for pathway-based gene data integration and visualization [23].

### 2.6. Quantitative Real-Time PCR (qRT-PCR)

Seven representative genes selected from DEGs involved in the neurotrophin-MAPK signaling pathway were validated by qRT-PCR in another 26 independent samples from each group (CN, DM, and DPN). Total RNA was extracted as described above, and double-stranded cDNA was synthesized using a PrimeScript RT Reagent Kit (TaKaRa Biotechnology, Dalian, China) according to the manufacturer's instructions. Real-time PCR was performed using LightCycler 480 SYBR Green I Master mix (Roche, Mannheim, Germany). The 2^−ΔΔCT^ method was used to quantify the relative expression of each gene, using GAPDH expression to normalize each sample. All experiments were run in triplicate and repeated three times. Differences in expression among groups were evaluated with a one-way analysis of variance (ANOVA) using SPSS 16.0 software. A significant difference was considered to be indicated by *P* < 0.05.

## 3. Results

### 3.1. Identification of DEGs among DM Patients, DPN Patients, and Healthy Controls

The gene expression data from this study are available in the Gene Expression Omnibus (GEO, accession number GSE95849). Compared with healthy individuals, patients with DM and DPN exhibited 2795 and 3606 upregulated genes, respectively, whereas 103 genes and 887 genes were expressed at a lower level in DM and DPN patients compared with the healthy controls (Table 2). Among these DEGs, DM and DPN patients shared 1947 upregulated and 95 downregulated genes. In DPN patients, compared with DM patients, 1942 genes were differentially expressed, including 931 upregulated and 1011 downregulated genes (Table 2).

### 3.2. Functional Annotation and Enrichment Analysis of the DEGs

**Figure 2:**
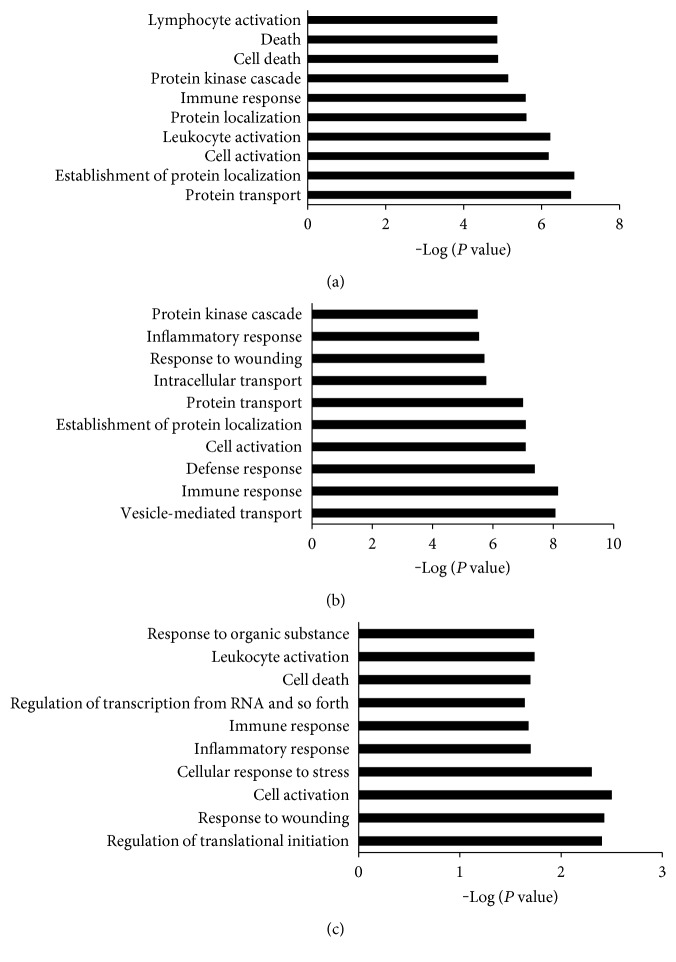
The top 10 biological processes identified by GO analysis of the DEGs: (a) DPN versus CN, (b) DM versus CN, and (c) DPN versus DM. Bars represent −log (*P* value).

According to the GO analysis, the DEGs of DM and DPN patients were enriched in many similar GO terms as well as distinct GO terms (Figure 2). Compared with the profile of healthy controls, the DM-associated gene expression profile primarily included DEGs related to intracellular transport, immune response, cell activation, protein localization, and inflammatory response (Figure 2(a)). The DPN patients exhibited differential expression of genes involved in protein transport, protein localization, leukocyte activation, immune response, protein kinase cascades, and cell death (Figure 2(b)). Between the DPN and DM group, the differentially expressed genes were enriched in cell activation, cellular response to stress, and cell death as well as in the regulation of transcription and translation (Figure 2(c)).

**Figure 3:**
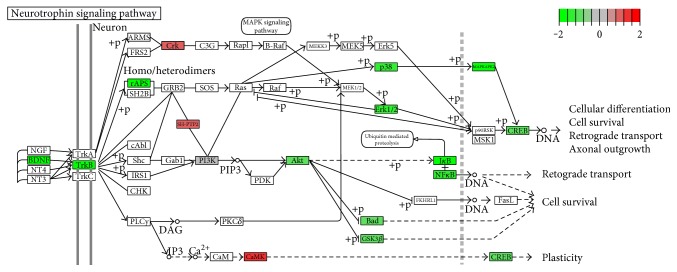
Downregulation of the neurotrophin-MAPK signaling pathway in DPN.

KEGG pathway analysis revealed that, in comparison with healthy controls, DPN and DM shared four pathways (Table 2), including apoptosis (hsa04210), B cell receptor signaling pathway (hsa04662), endocytosis (hsa04144), and Toll-like receptor signaling pathway (hsa04620). Moreover, Fc gamma R-mediated phagocytosis (hsa04666), chemokine signaling pathway (hsa04062), and insulin signaling pathway (hsa04910) were enriched in the DM group, and NOD-like receptor signaling pathway (hsa04621), lysosome (hsa04142), valine, leucine and isoleucine degradation (hsa00280), and amino sugar and nucleotide sugar metabolism (hsa00520) were enriched in the DPN group. In contrast, for DEGs between DPN and DM patients, the most enriched pathway was MAPK signaling pathway (hsa04010), followed by NOD-like receptor signaling pathway (hsa04621) and neurotrophin signaling pathway (hsa04722). For the gene set lists of these three pathways, see electronic Supplementary Table 1 available online at https://doi.org/10.1155/2017/8103904. When the DEGs identified between DPN and DM patients were further analyzed with regard to upregulation and downregulation, the upregulated genes were enriched in the infectious disease category, whereas the downregulated genes were enriched in the “neurotrophin signaling pathway” and “MAPK signaling pathway” (Table 3). A KEGG Pathview analysis also showed that downregulation of the neurotrophin-MAPK signaling pathway was the major pathogenesic pathway in diabetic peripheral neuropathy patients (Figure 3()).

### 3.3. Validation of DEGs in the Neurotrophin-MAPK Signaling Pathway

**Figure 4:**
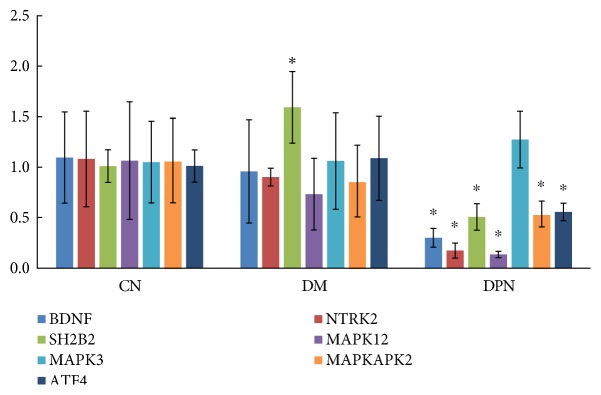
Microarray data were validated by qRT-PCR. The results are expressed as the mean ± SD. The asterisks represent statistical significance (*P* < 0.05) compared with the control group.

Seven DEGs selected from the “neurotrophin-MAPK signaling pathway” were further validated in an additional 78 participants by qRT-PCR. These genes are *BDNF* (brain-derived neurotrophic factor), *NTRK2* (neurotrophic tyrosine kinase type 2 receptor, also known as TrkB), *SH2B2* (SH2B adaptor protein 2, also known as rAPS), *MAPK3* (mitogen-activated protein kinase 3, also known as Erk), *MAPK12* (mitogen-activated protein kinase 12, also known as p38), *MAPKAPK2* (mitogen-activated protein kinase-activated protein kinase 2), and *ATF4* (activating transcription factor 4, also known as CREB). The results confirmed the initial microarray findings for all of the genes except *MAPK3*, whose expression was not changed in DM patients or in DPN patients (Figure 4).

## 4. Discussion

DPN is the most common complication of diabetes, affecting up to 50% of patients, and it contributes significantly to pain, loss of sensation, numbness, injury, and lower extremity amputation [4]. Because complex pathways are implicated in the pathophysiology of DPN, there are still no specific treatments and no means of predicting or preventing DPN onset or progression. Gene expression microarrays have been widely used in diabetic studies, because alterations in transcriptional profiles provide a robust and sensitive way to better understand the mechanisms of the disease and its complications. Microarray analyses have previously been used to investigate the mechanisms underlying diabetic cardiomyopathy [24], diabetic nephropathy [25], diabetic bone disease [26], and diabetic periodontitis [27] in diabetic patients or animal models. However, only one study has been conducted in patients with diabetic neuropathy to identify the DEGs and the related biological pathways responsible for the progression of diabetic neuropathy [28]. That study comprised a microarray experiment performed on human sural nerves collected from 50 patients with diabetic neuropathy during a 52-week clinical trial. Hyperglycemia may cause damage to the majority of the peripheral nerve system, not just the sural nerve. Moreover, as mentioned in that article, it is highly unlikely that future studies of diabetic neuropathy in patients will include the collection of sural nerve biopsies [28]. Therefore, performing new transcriptional microarray analyses with commonly used human sample material, such as PBMCs, to identify the gene expression signatures of DPN is necessary. Actually, transcriptome studies in PBMCs have been widely used in diabetic studies. Gene expression profiles in PBMCs can clinically stratify patients with recent-onset type 1 DM [29] and reflect the pathophysiology of type 2 DM [30]. In PBMCs from children with diabetes, the gene expression microarrays identified that type 1 and type 2 DM likely shared a common pathway for *β*-cell dysfunction that includes secretion of IL-1*β* and prostaglandins by immune effector cells [31]. Furthermore, a microarray study of gene expression in PBMCs identified that *THBS1* and *COX1* genes were upregulated, while *MMP9* and *COX2* genes were downregulated in patients with diabetic nephropathy [32].

The abovementioned microarray study on DPN identified DEGs that are functionally enriched in inflammatory responses and defense response pathways and may potentially be responsible for the progression of diabetic neuropathy [28]. In the current study, we found that DPN and DM shared four pathways, at least two of which were directly associated with immune-related functions, the “B cell receptor signaling pathway” and “Toll-like receptor signaling pathway.” It is widely accepted that inflammation and immunity are crucially involved in diabetes and a majority of diabetic complications [33, 34]; therefore, changes in the immune response may not be a distinct mechanism of DPN. We further compared the expression profiles of DPN and DM patients and identified that DPN-specific DEGs were significantly enriched in “MAPK signaling pathway”, “NOD-like receptor signaling pathway”, and “neurotrophin signaling pathway”. A stratification analysis further indicated that “neurotrophin signaling pathway” and “MAPK signaling pathway” were the top two pathways with downregulated DEGs. Although the DEGs in neurotrophin-MAPK signaling pathway are all located in autosome, caution should still be taken since only female subjects were involved in the present study, and more experiments are required to confirm it in male patients.

Neurotrophic factors are essential molecules that develop and maintain the nervous system by promoting the growth and survival of neurons. The neurotrophin family of growth factors includes nerve growth factor (NGF), brain-derived neurotrophic factor (BDNF), and neurotrophin- (NT-) 3/4/5. Glial cell-derived neurotrophic factors (GDNF) form a second family and include GDNF, neurturin, artemin, and persephin [35, 36]. Deficiencies in NGF and NT-3 have long been reported in both the tissue and sera of DPN patients. Diabetes also decreases the anterograde and retrograde axonal transport of BDNF, NGF, and NT-3 in peripheral nerves [37–39]. In the current study, the expression levels of NGF, NT-3, and NT-4 remained unchanged. However, the BDNF level decreased significantly. BDNF plays important roles in regulating the survival and growth of neurons and influences synaptic efficiency and plasticity. The human BDNF gene consists of 11 exons, and the majority of the BDNF transcripts are detected not only in the brain but also in blood cells [40]. BDNF mediates neuronal differentiation and survival by binding and activating tropomyosin receptor kinase B (TrkB), an important member of the larger Trk family [41]. In the current study, the *NTRK2* gene, which encodes TrkB in humans, was specifically downregulated in DPN patients but not in DM patients, thus strongly suggesting that downregulation of the BDNF-TrkB signaling pathway is associated with DPN.

The binding of BDNF to TrkB leads to the dimerization and autophosphorylation of tyrosine residues in the intracellular domain of the receptor [42], which in turn leads to phosphorylation of tyrosine residues in the juxtamembrane domain or the C-terminus of the receptor [43]. These tyrosine residues serve as docking sites for multiple adaptor molecules, including Shc adaptor proteins, fibroblast growth factor receptor substrate 2 (FRS2), phospholipase C*γ* (PLC*γ*), SH2B adaptor proteins, ankyrin repeat-rich membrane spanning (ARMS), Csk homology kinase (CHK), insulin receptor substrate 1 (IRS1), and c-ABL1 [43]. Among these adaptor molecules, only the expression level of *SH2B2* (also known as rAPS) decreased in DPN patients, probably due to the downregulation of TrkB. TrkB activates three major intracellular signaling cascades: the mitogen-activated protein kinase (MAPK) pathway, phosphatidylinositol 3-kinase (PI3K)-Akt pathway, and the PLC*γ*-Ca^2+^ pathway [44]. However, in the current study, only the MAPK pathway was affected in DPN patients. Among the three major MAPK family members, the Erk1/2 and p38 pathways were found to be downregulated in our original microarray study. However, after qRT-PCR validation, only the expression level of p38 decreased. This result is actually reasonable, because the direct downstream target of p38, MAPKAPK2, was also downregulated, whereas the downstream targets of Erk1/2, MSK1, and p90RSK were unchanged.

In neuronal development and function, neurotrophins activate the p38 MAPK/MAPKAPK2 pathway through Trk receptor-mediated signaling mechanisms [45]. Subsequently, MAPKAPK2 phosphorylates CREB (cAMP response element binding protein) and other transcription factors [46]. These transcription factors in turn regulate the expression of genes whose products are involved in many aspects of neural development and function, including cell fate decisions, axon growth, dendrite pruning, synaptic function, and plasticity [45]. In the present study, 2898 genes exhibited altered expression in DM patients, whereas this number in DPN patients was 4493. Moreover, in DPN patients, compared with DM patients, the number of downregulated genes increased 7 times, possibly because of decreased CREB expression mediated by the downregulation of the neurotrophin-MAPK signaling pathway.

In addition to the “neurotrophin signaling pathway” and “MAPK signaling pathway,” the present study also showed that the “NOD-like receptor signaling pathway” was affected in DPN patients. Nucleotide-binding oligomerization domain containing 2 (NOD2), a member of the NOD-like receptor family, has been found to be one of the critical components of a signal transduction pathway linking renal injury to inflammation and podocyte insulin resistance in diabetic nephropathy [47]. A recent study has also suggested that the formation and activation of the NOD-like receptor protein 1 (NLRP1) inflammasome induces neuroinflammation and neuron injury during hyperglycemia, thus representing a novel mechanism of diabetes-associated neuron injury [48]. Interestingly, our microarray data indicated that the expression of *NOD2* and *NLRP1* remained unchanged in DM patients but increased significantly in DPN patients (3.76-fold for *NOD2* and 2.07-fold for *NLRP1*). Therefore, the NOD-like receptor signaling pathway may be an important mechanism in the pathogenesis of diabetic complications.

It is now widely accepted that increased inflammation is a key etiological factor in the development of many chronic diseases, including diabetes [49]. Similar to Nod-like receptors, Toll-like receptors (TLRs) belong to another major family of pattern recognition receptors, which have been demonstrated to play a critical role in the innate immune system [50]. Among these TLRs, TLR4 plays an important role in many inflammatory disorders, and system inflammation facilitated by TLR4 is involved in the pathophysiological process of diabetes [51]. A recent study has further demonstrated that TLR4 may be a potential diagnostic biomarker for DPN [52]. In the current microarray study, *TLR4* expression increased in both DM and DPN patients, and there was even higher expression in the DPN patients (Table 4), in accordance with the results of the abovementioned study [52]. Moreover, it has been suggested that the inflammatory effects of high glucose may be mediated through the modulation of inflammatory responses resulting from TLR activation in diabetes [53]. The present microarray study showed that the “Toll-like receptor signaling pathway” was significantly enriched, and all TLR family genes were upregulated in both DPN and DM patients (Tables 4 and 5). These results strongly suggest that although some TLR genes, such as *TLR4* and *TLR7*, were expressed at much higher levels in DPN patients, they may not be sensitive biomarkers for DPN, because the TLR signaling pathway is involved in diabetes and multiple diabetic complications.

## 5. Conclusions

In summary, these findings provide the first demonstration that downregulation of the neurotrophin-MAPK signaling pathway may be the major pathogenesis of DPN. The use of growth factors in treating DPN has been extensively explored [54], and NGF, BDNF, and NT-3 have been assessed in various levels of clinical trials of DPN, with limited success [35]. The current study suggests that pharmacological targeting of both the neurotrophin signaling pathway and the MAPK signaling pathway at multiple levels may provide a potential approach for the treatment of DPN.

## Supplementary Material

Supplementary Table S1: Gene set lists of three enriched KEGG pathways.

## Figures and Tables

**Table 1:** Clinical and demographic characteristics of the participants.

	Control	T2DM	DPN
Discovery panel			
Number	6	6	6
Gender	Female	Female	Female
Age (y)	51.17 ± 6.08	53.83 ± 10.80	57.33 ± 7.92
Fasting glucose (mmol/L)	5.10 ± 0.21	9.23 ± 2.02^∗^	9.95 ± 2.11^∗^
Validation panel			
Number	26	26	26
Gender	Female	Female	Female
Age (y)	50.91 ± 5.60	51.25 ± 8.13	53.21 ± 11.15
Fasting glucose (mmol/L)	5.27 ± 0.59	8.96 ± 1.58^∗^	9.72 ± 1.81^∗^

The asterisks represent statistical significance (*P* < 0.05) compared with the control group.

**Table 2:** Number of differentially expressed genes in different groups.

	DM versus CN	DPN versus CN	DPN versus DM
Upregulated	2795	3606	931
Downregulated	103	887	1011

**Table 3:** The significant KEGG pathways for the DEGs of DPN versus DM.

	KEGG pathway	Count	Benjamini *q* value
Upregulated	hsa05164: influenza A	25	0.001
	hsa05162: measles	21	0.001
	hsa00020: citrate cycle (TCA cycle)	9	0.008
	hsa05168: herpes simplex infection	23	0.009

Downregulated	hsa04722: neurotrophin signaling pathway	21	0.001
	hsa04010: MAPK signaling pathway	31	0.003
	hsa04668: TNF signaling pathway	18	0.003
	hsa04380: osteoclast differentiation	20	0.003
	hsa05169: Epstein-Barr virus infection	25	0.003
	hsa05166: HTLV-I infection	30	0.003
	hsa04660: T cell receptor signaling pathway	16	0.010
	hsa05215: prostate cancer	14	0.020
	hsa05142: Chagas disease (American trypanosomiasis)	15	0.029
	hsa05145: toxoplasmosis	16	0.031
	hsa04068: FoxO signaling pathway	17	0.039
	hsa04064: NF-kappa B signaling pathway	13	0.039
	hsa04621: NOD-like receptor signaling pathway	10	0.040

**Table 4:** Expression of TLR family genes.

Gene symbol	Log_2_ (ratio)			*P* value		
	DM/CN	DPN/CN	DPN/DM	DM/CN	DPN/CN	DPN/DM
*TLR1*	1.744	2.033	0.289	4.40*E*−04	5.22*E*−05	0.516
*TLR2*	2.175	2.056	−0.119	9.34*E*−04	1.05*E*−05	0.818
*TLR4*	2.861	4.254	1.393	1.73*E*−05	5.71*E*−09	1.38*E*−02
*TLR5*	2.213	3.682	1.469	6.31*E*−03	2.55*E*−11	0.054
*TLR6*	2.908	2.169	−0.738	4.24*E*−05	5.15*E*−06	0.213
*TLR7*	1.677	3.475	1.799	1.44*E*−02	2.72*E*−09	6.67*E*−03
*TLR8*	2.041	2.785	0.745	1.31*E*−04	2.61*E*−06	0.109
*TLR9*	1.623	1.135	−0.488	3.37*E*−04	3.82*E*−04	0.232

*TLR3* and *TLR10* were removed after data normalization because of the low fluorescent intensity of probes.

**Table 5:** The significant KEGG pathways for the DEGs of different groups.

	KEGG pathway	Count	Benjamini *q* value
DM	hsa04210: apoptosis	32	0.009
	hsa04620: Toll-like receptor signaling pathway	35	0.007
	hsa04144: endocytosis	53	0.014
	hsa04666: Fc gamma R-mediated phagocytosis	32	0.014
	hsa04062: chemokine signaling pathway	53	0.013
	hsa04662: B cell receptor signaling pathway	26	0.024
	hsa04910: insulin signaling pathway	39	0.044

DPN	hsa04662: B cell receptor signaling pathway	39	0.000
	hsa04210: apoptosis	40	0.004
	hsa04621: NOD-like receptor signaling pathway	31	0.004
	hsa04144: endocytosis	69	0.009
	hsa04620: Toll-like receptor signaling pathway	41	0.032
	hsa04142: lysosome	46	0.028
	hsa00280: valine, leucine, and isoleucine degradation	22	0.026
	hsa00520: amino sugar and nucleotide sugar metabolism	22	0.026

DPN versus DM	hsa04010: MAPK signaling pathway	53	0.001
	hsa04621: NOD-like receptor signaling pathway	18	0.016
	hsa04722: neurotrophin signaling pathway	27	0.029

## Abbreviations

BDNF: Brain-derived neurotrophic factor
CREB: cAMP response element binding protein
DAVID: Database for Annotation, Visualization and Integrated Discovery
DEG: Differentially expressed gene
DM: Diabetes mellitus
DPN: Diabetic peripheral neuropathy
GO: Gene Ontology
KEGG: Kyoto Encyclopedia of Genes and Genomes
NGF: Nerve growth factor
OGTT: Oral glucose tolerance test
qRT-PCR: Quantitative real-time PCR
RIN: RNA integrity number
TLR: Toll-like receptor.
